# Supplementary material for: Arabidopsis R1R2R3-Myb proteins are essential for inhibiting cell division in response to DNA damage
Source: Nat Commun. 2017 Sep 21;8:635. doi: 10.1038/s41467-017-00676-4 (PMC5608833; doi:10.1038/s41467-017-00676-4)
Supplement: Supplementary file 1 — Supplementary Information [file 41467_2017_676_MOESM1_ESM.pdf]

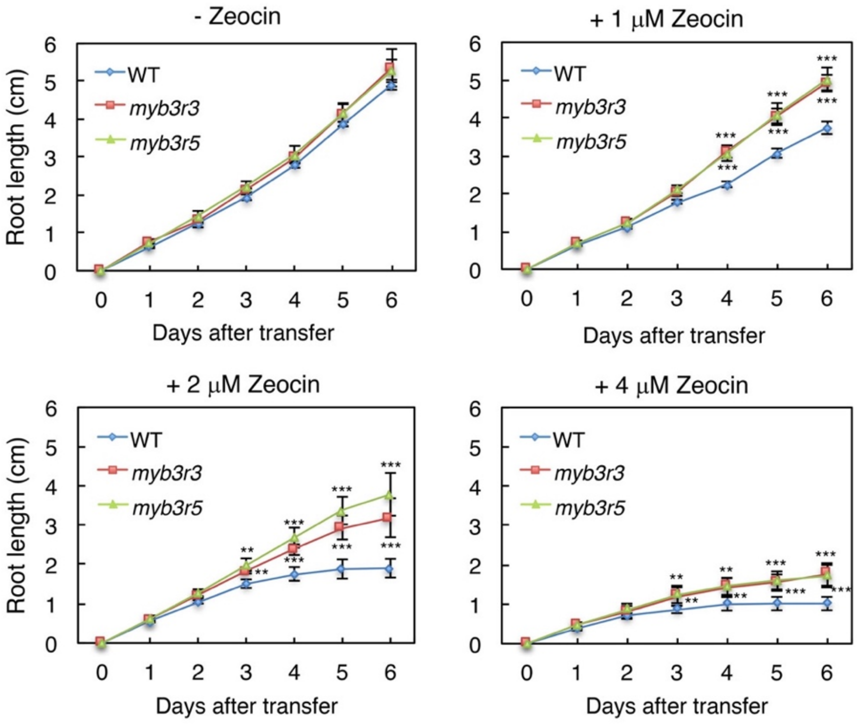


**Supplementary Figure 1.** Root growth of *myb3r3* and *myb3r5* in the presence of different concentrations of zeocin.

Five-day-old seedlings of WT, *myb3r3* and *myb3r5* were transferred to medium with or without 1, 2 or 4 μM zeocin, and root length was measured for 6 days. Data are presented as mean ± SD (n = 30). Significant differences from WT were determined by Student's *t*-test: **, $P < 0.01$; ***, $P < 0.001$.

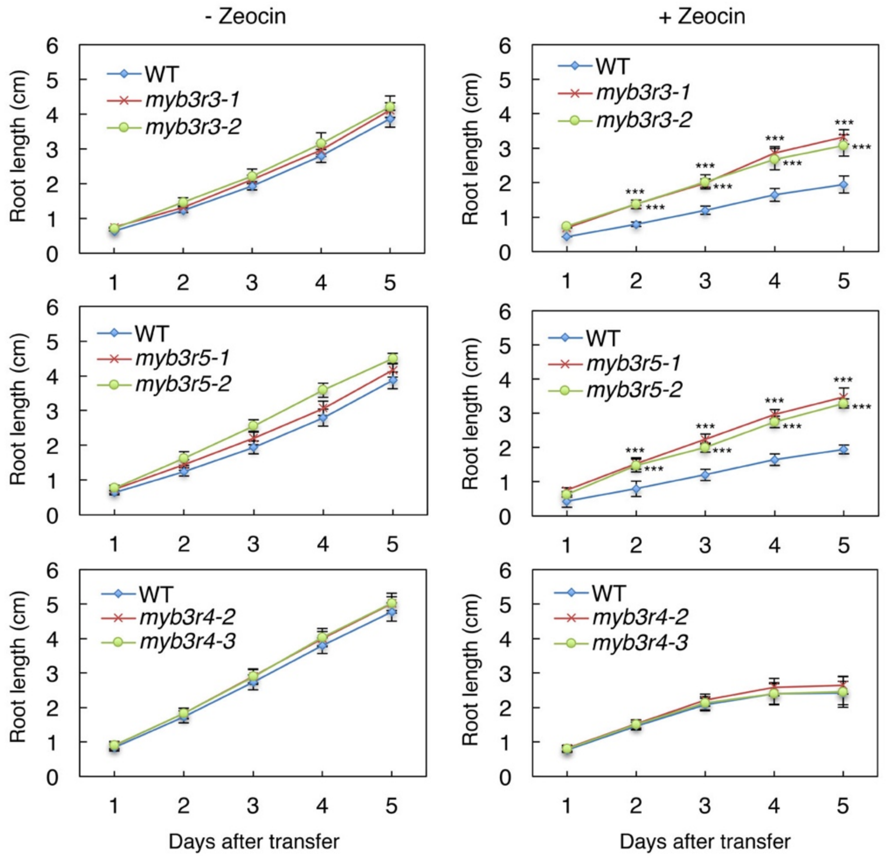


**Supplementary Figure 2.** Root growth of other *myb3r* mutants.

Five-day-old seedlings were transferred to medium with or without 2 μM zeocin, and root length was measured for 5 days. Data are presented as mean ± SD (n = 30). Significant differences from WT were determined by Student's *t*-test: ***, $P < 0.001$.

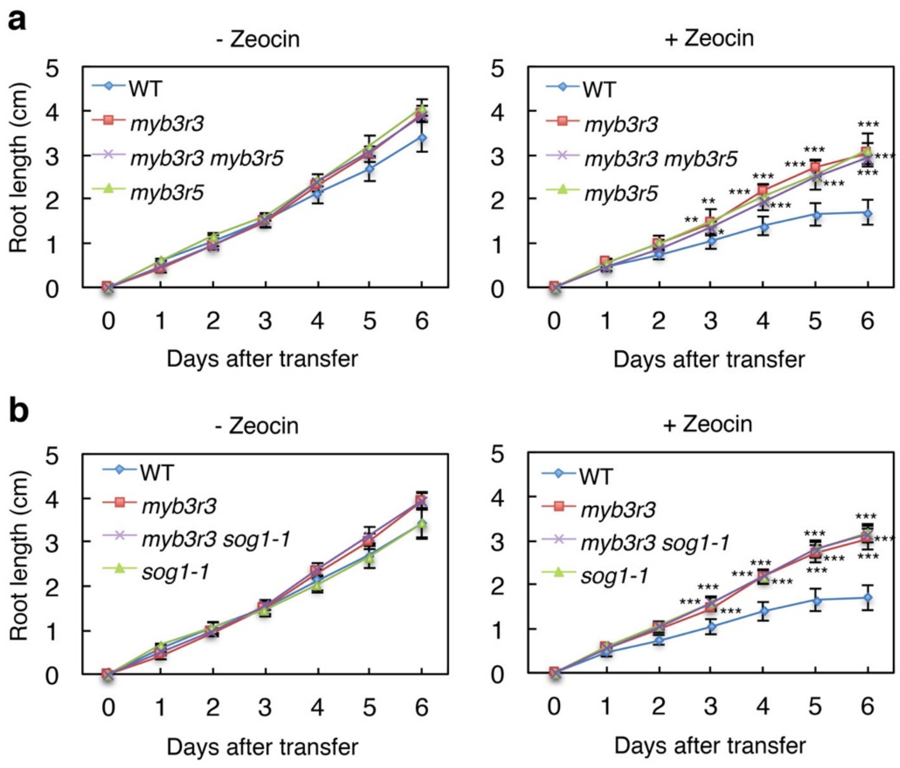


**Supplementary Figure 3.** Root growth of *myb3r3 myb3r5* and *myb3r3 sog1-1*.

Five-day-old seedlings were transferred to medium with or without 2 μM zeocin, and root length was measured to estimate the zeocin response of *myb3r3 myb3r5* (**a**) and *myb3r3 sog1-1* (**b**). Data are presented as mean ± SD (n = 30). Significant differences from WT were determined by Student's *t*-test: *, $P < 0.05$; **, $P < 0.01$; ***, $P < 0.001$.

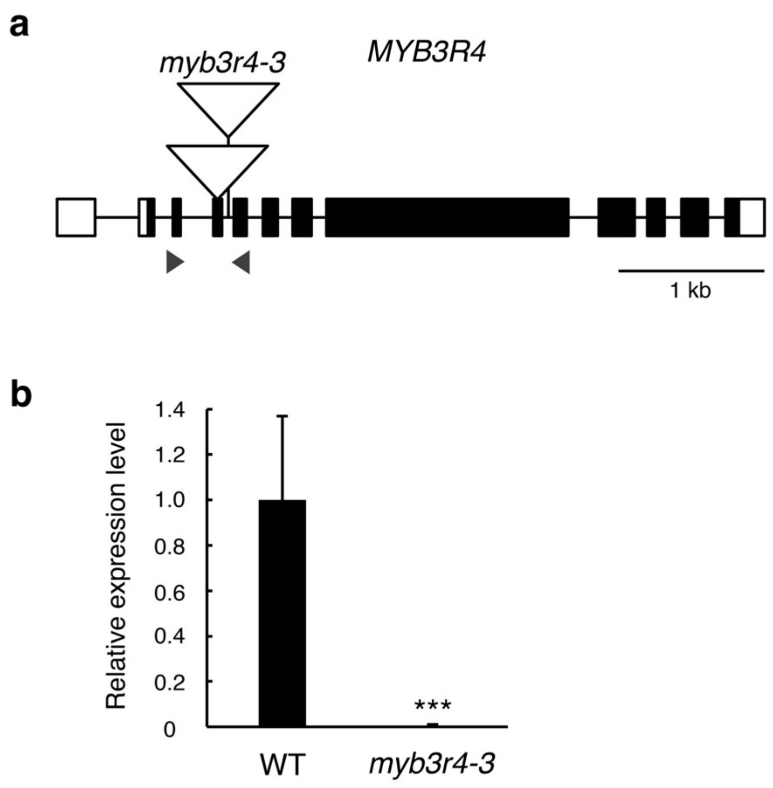


**Supplementary Figure 4.** Characterization of the *myb3r4-3* allele.

(**a**) T-DNA insertion sites in *myb3r4-3*. Exons and introns are indicated by boxes and solid lines, respectively. Protein-coding regions are shown by black boxes. Black triangles indicate the sites of primers used in (**b**). Note that T-DNA is inserted into exon 4 and intron 4. (**b**) Quantitative RT-PCR analysis of *MYB3R4*. Total RNA was isolated from two-week-old WT and *myb3r4-3* seedlings, and subjected to quantitative RT-PCR using primers shown in (**a**). The expression levels were normalized to that of *ACTIN2*, and are indicated as relative values, with that of WT set to 1. Data are presented as mean ± SD of three biological replicates. Significant difference from WT was determined by Student's *t*-test: ***, $P < 0.001$.

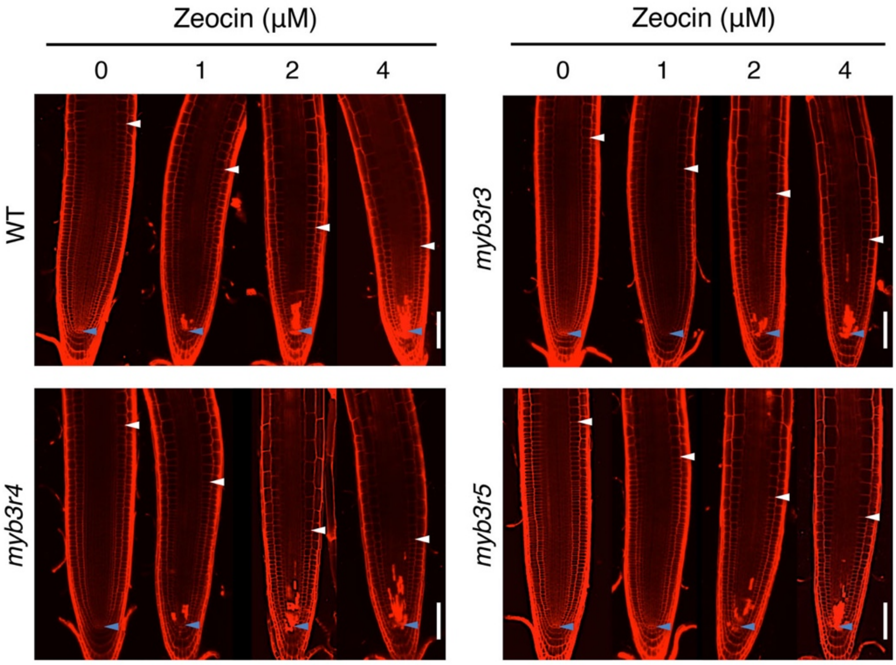


**Supplementary Figure 5.** Confocal microscopy images of root tips of *myb3r* mutants.

Five-day-old seedlings were treated with or without 1, 2 or 4 μM zeocin for 24 h, and root tips were observed after staining with propidium iodide. Blue and white arrowheads indicate the quiescent centre and the first elongated cell in the cortex cell file, respectively. Scale bars, 100 μm.

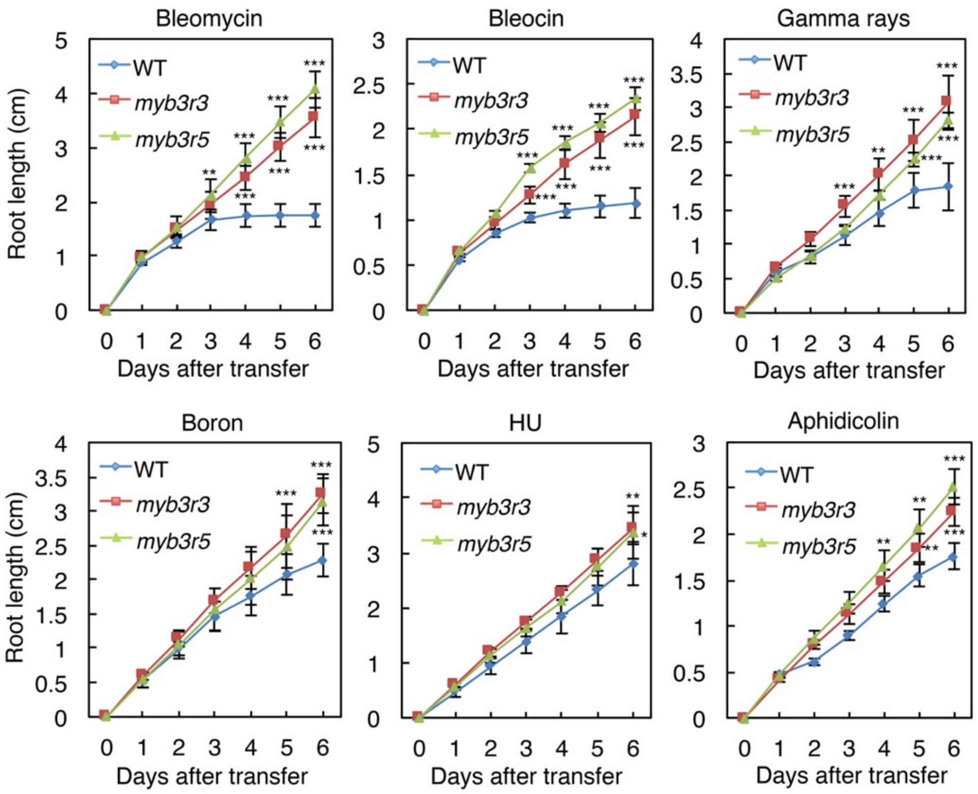


**Supplementary Figure 6.** Response of *myb3r3* and *myb3r5* to various types of DNA damage.
Five-day-old seedlings of WT, *myb3r3* and *myb3r5* were transferred to medium containing 0.25 μg $ml^{-1}$ bleomycin, 0.06 μg $ml^{-1}$ bleocin, 5 mM boron, 1 mM hydroxyurea (HU) or 5 μg $ml^{-1}$ aphidicolin, and root length was measured for 6 days. Root growth was also observed after irradiation with gamma rays (100 Gy). Data are presented as mean ± SD (n > 30). Significant differences from WT were determined by Student's *t*-test: *, $P < 0.05$; **, $P < 0.01$; ***, $P < 0.001$.

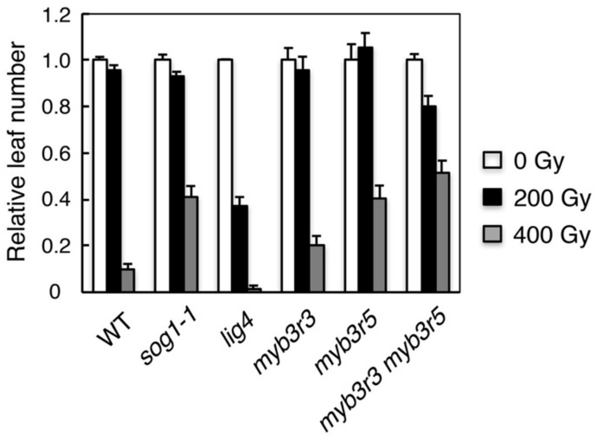


**Supplementary Figure 7.** Leaf production of *myb3r* mutants after gamma irradiation.

Seeds were irradiated with gamma rays (200 or 400 Gy), and the number of true leaves was counted using 10-day-old seedlings. The numbers of true leaves are expressed as relative values, with that of the non-treated seedlings set to 1. Data are presented as mean ± SD (n > 80). *sog1-1* and *lig4* were used as controls.

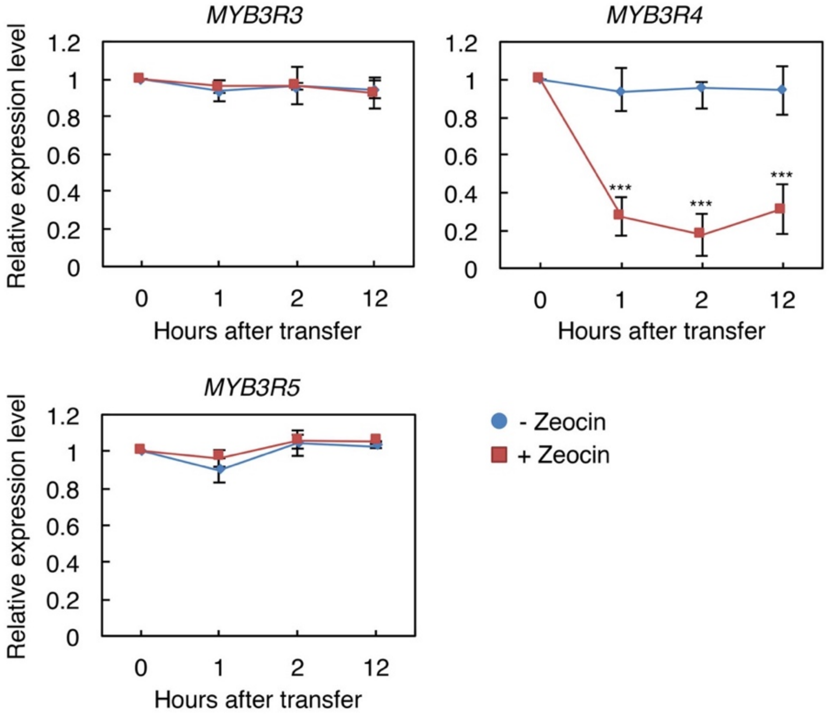


**Supplementary Figure 8.** Quantitative RT-PCR analysis of *MYB3R* genes in the presence of zeocin. Five-day-old WT seedlings were transferred to medium with or without 2 μM zeocin, and grown for the indicated times. Total RNA was isolated from a 1-cm region at the root tip. Expression levels were normalized to that of *ACTIN2*, and are indicated as relative values, with that for the time of transfer to new medium set to 1. Data are presented as mean ± SD of three biological replicates. Significant differences from the control without zeocin were determined by Student's *t*-test: ***, $P < 0.001$.

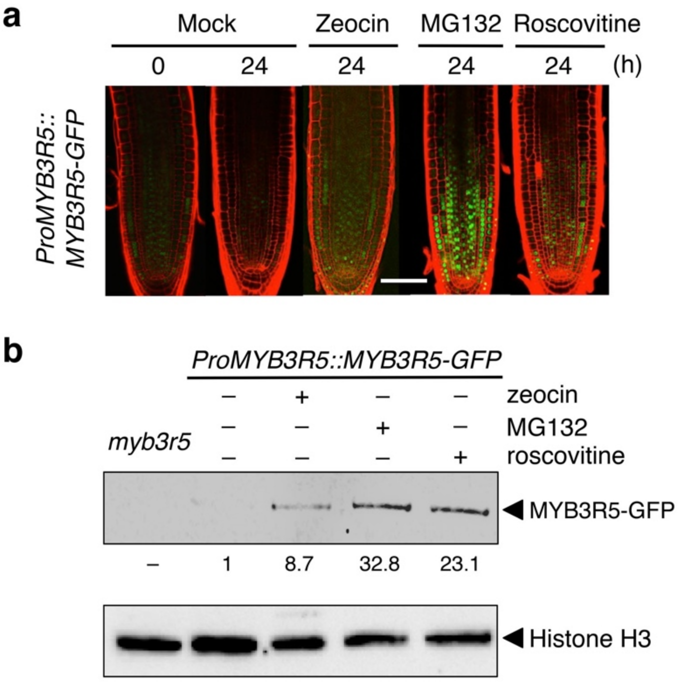


**Supplementary Figure 9.** MYB3R5 accumulation in the root tip.

(**a**) Confocal microscopy images of root tips. Five-day-old *myb3r5* seedlings harbouring *ProMYB3R5::MYB3R5-GFP* were treated with or without 2 μM zeocin, 50 μM MG132 or 25 μM roscovitine for 24 h. Scale bar, 100 μm. (**b**) Protein level of MYB3R5-GFP. Ten-day-old *myb3r5* seedlings harbouring *ProMYB3R5::MYB3R5-GFP* were treated with or without 2 μM zeocin, 50 μM MG132 or 25 μM roscovitine for 24 h, and 40 μg of total protein extracted from roots was immunoblotted with anti-GFP or anti-histone H3 antibodies. Protein extract from the *myb3r5* mutant was used as a control. Relative levels of MYB3R5-GFP are expressed as the fold change, normalized with respect to the band of histone H3.

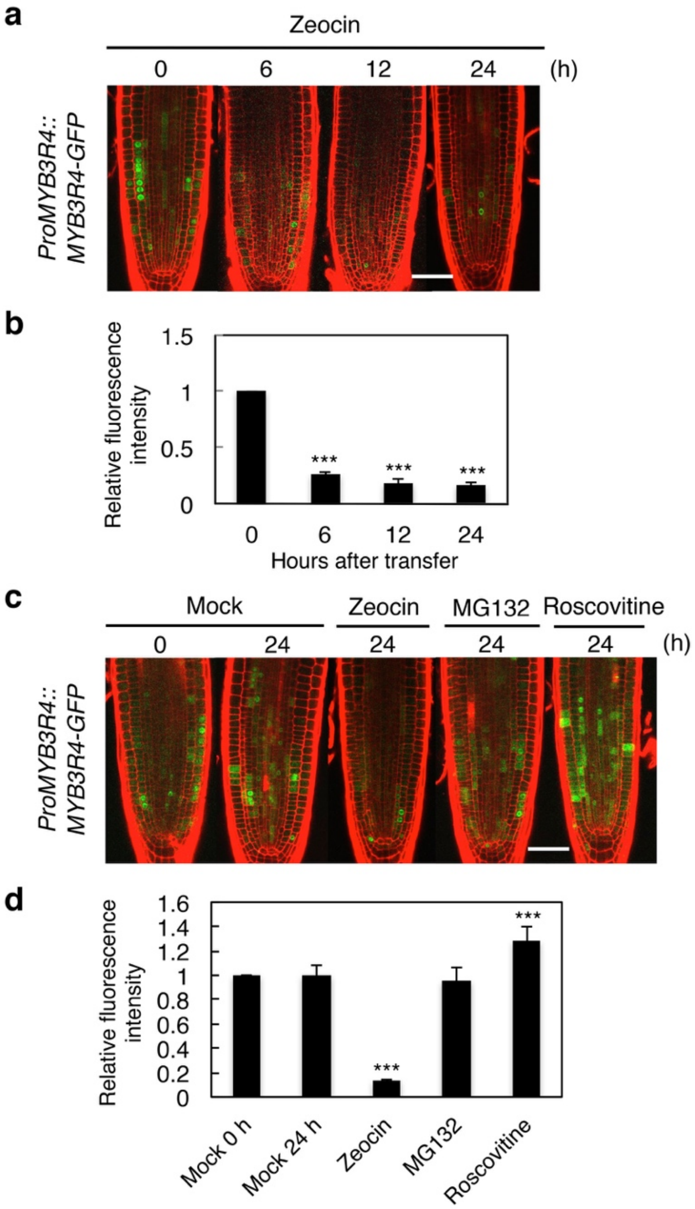


**Supplementary Figure 10.** MYB3R4 accumulation in the root tip.

(**a**, **c**) Confocal microscopy images of root tips. Five-day-old *myb3r4* seedlings harbouring *ProMYB3R4::MYB3R4-GFP* were treated with 2 μM zeocin for the indicated times (**a**), or with or without 2 μM zeocin, 50 μM MG132 or 25 μM roscovitine for 24 h (**c**). Scale bars, 100 μm. (**b**, **d**) Fluorescence intensity of MYB3R4-GFP shown in (**a**) and (**c**), respectively. Fluorescence intensities in the root tip are indicated as relative values, with that for the time of transfer to new medium with or without zeocin, MG132 or roscovitine set to 1. Data are presented as mean ± SD (n = 10). Significant differences from that for the time of transfer were determined by Student's *t*-test: ***, $P < 0.001$.

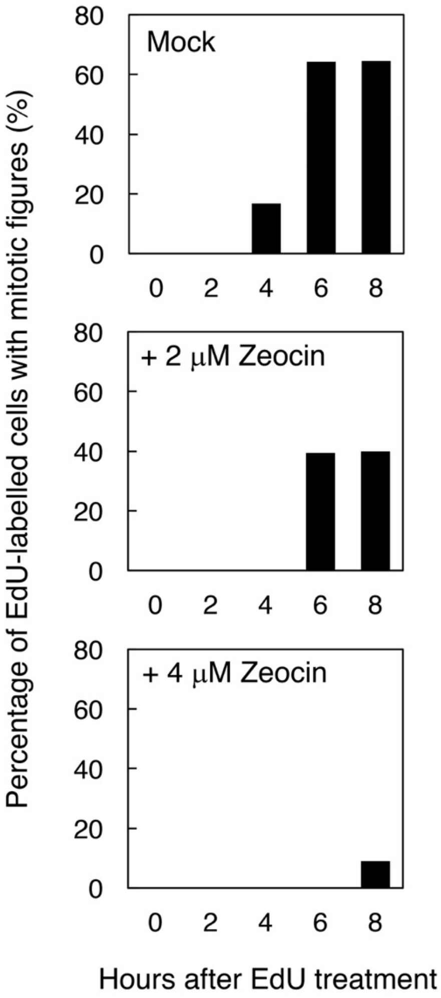


**Supplementary Figure 11.** Inhibition of entry into M phase by zeocin treatment.
Four-day-old WT seedlings were grown on medium with or without 2 or 4 μM zeocin for 12 h, and pulse-labelled with EdU. Seedlings were then transferred back to medium with or without zeocin, and collected after the indicated time points. Root meristematic cells were double-stained with EdU and DAPI, and cells with mitotic figures were counted. Data are presented as the percentage of EdU-labelled cells among those with mitotic figures (n > 22).

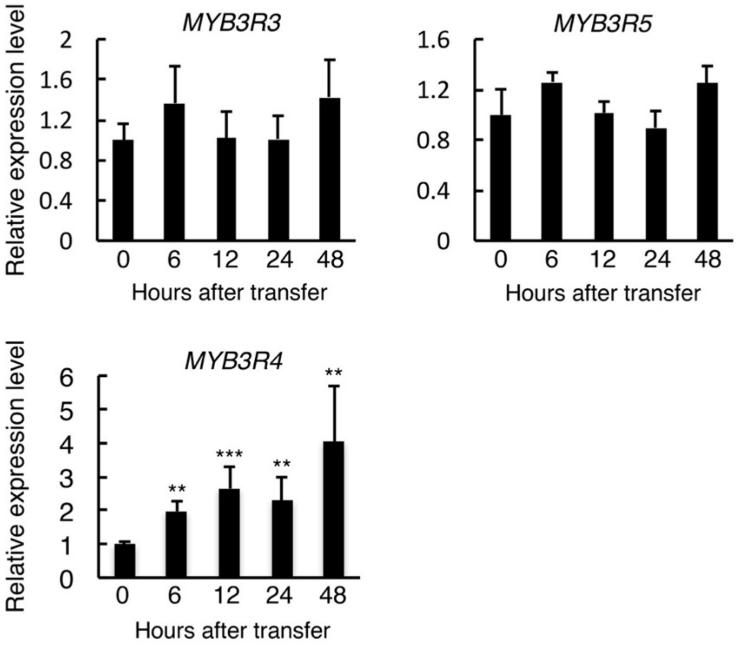


**Supplementary Figure 12.** Quantitative RT-PCR analysis of *MYB3R* genes in the presence of roscovitine. Five-day-old WT seedlings were transferred to medium with 25 μM roscovitine, and grown for the indicated times. Total RNA was isolated from a 1-cm region at the root tip. Expression levels were normalized to that of *ACTIN2*, and are indicated as relative values, with that for the time of transfer to roscovitine-containing medium set to 1. Data are presented as mean ± SD of three biological replicates. Significant differences from that for the time of transfer were determined by Student's *t*-test: **, $P < 0.01$; ***, $P < 0.001$.

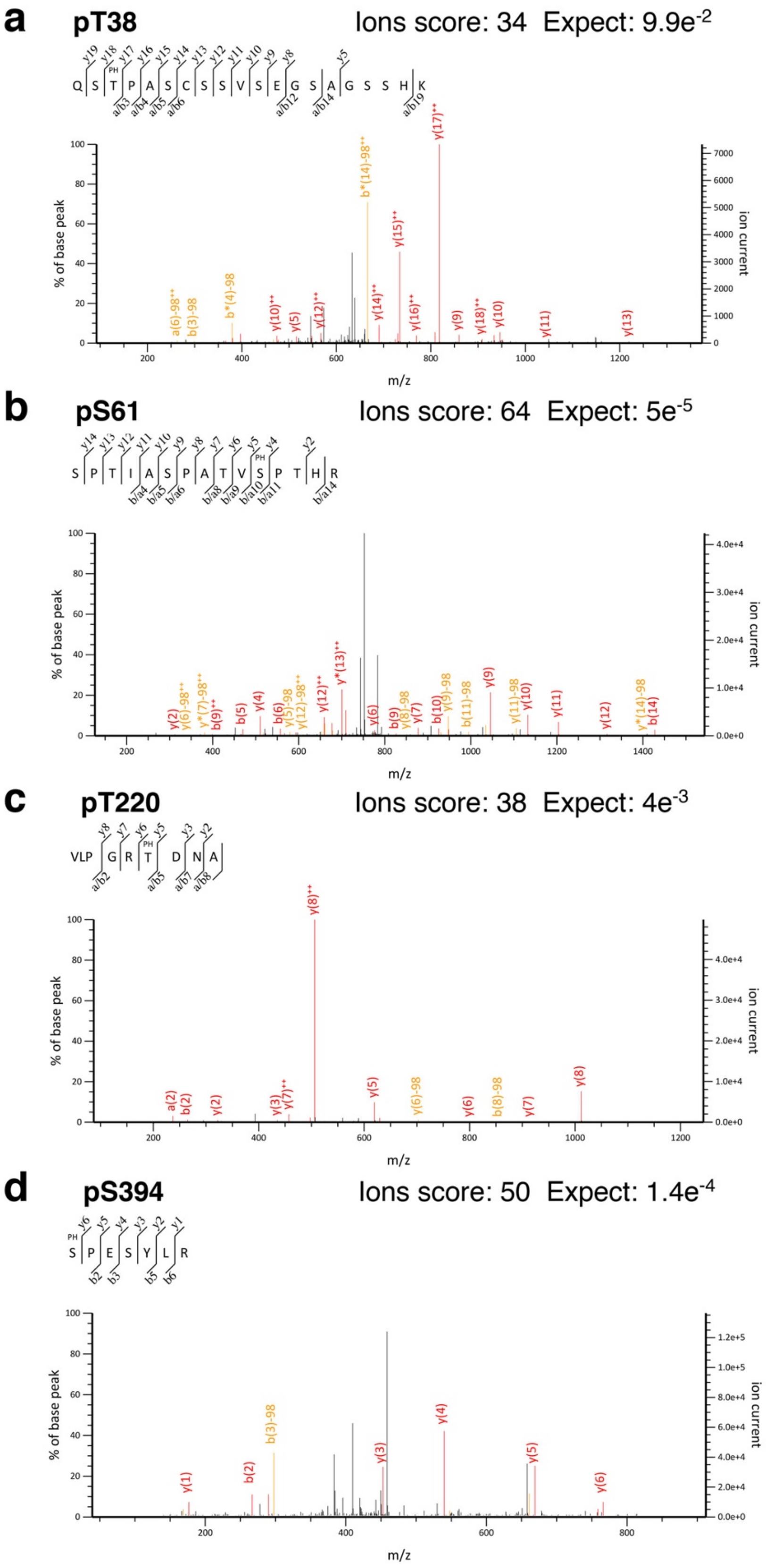


*Continued on next page*

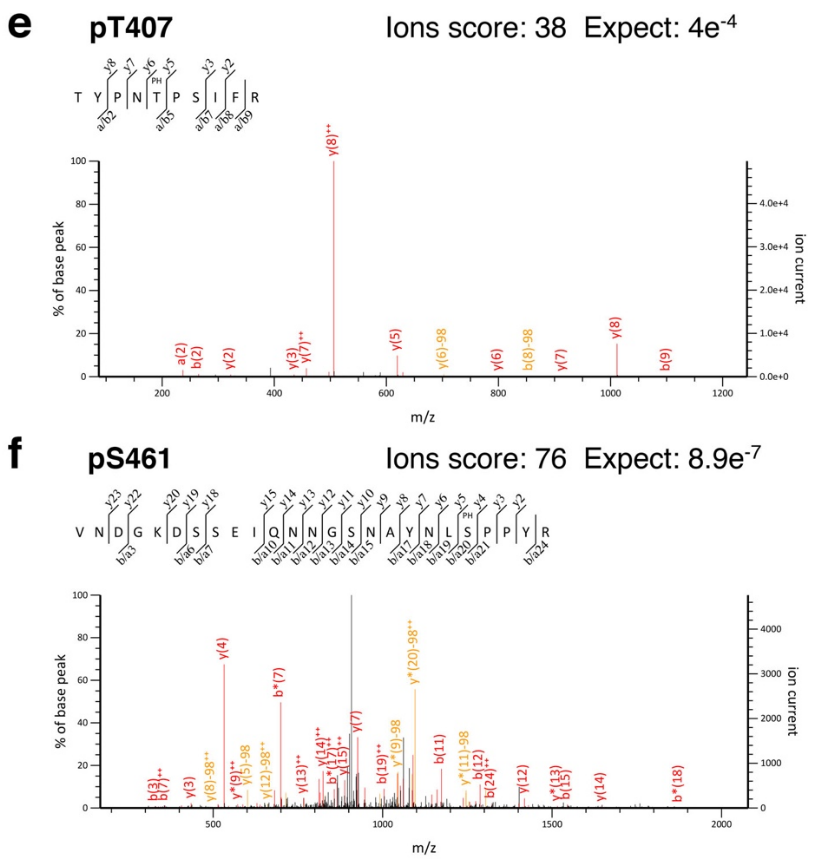


**Supplementary Figure 13.** Identification of phosphorylation sites of MYB3R3 by mass spectrometry. GST-MYB3R3 phosphorylated by CDKA;1-CYCD3;1 complexes was separated by SDS-PAGE and in-gel-digested with trypsin, followed by LC-MS/MS analysis. MS/MS spectra representing phosphorylation at Thr38 (**a**) Ser61 (**b**), Thr220 (**c**), Ser394 (**d**), Thr407 (**e**) and Ser461 (**f**) are shown. Ions score indicates values calculated using Mascot software. Peptides with an ions score ≥ 30 and an E-value < 0.05 are shown.

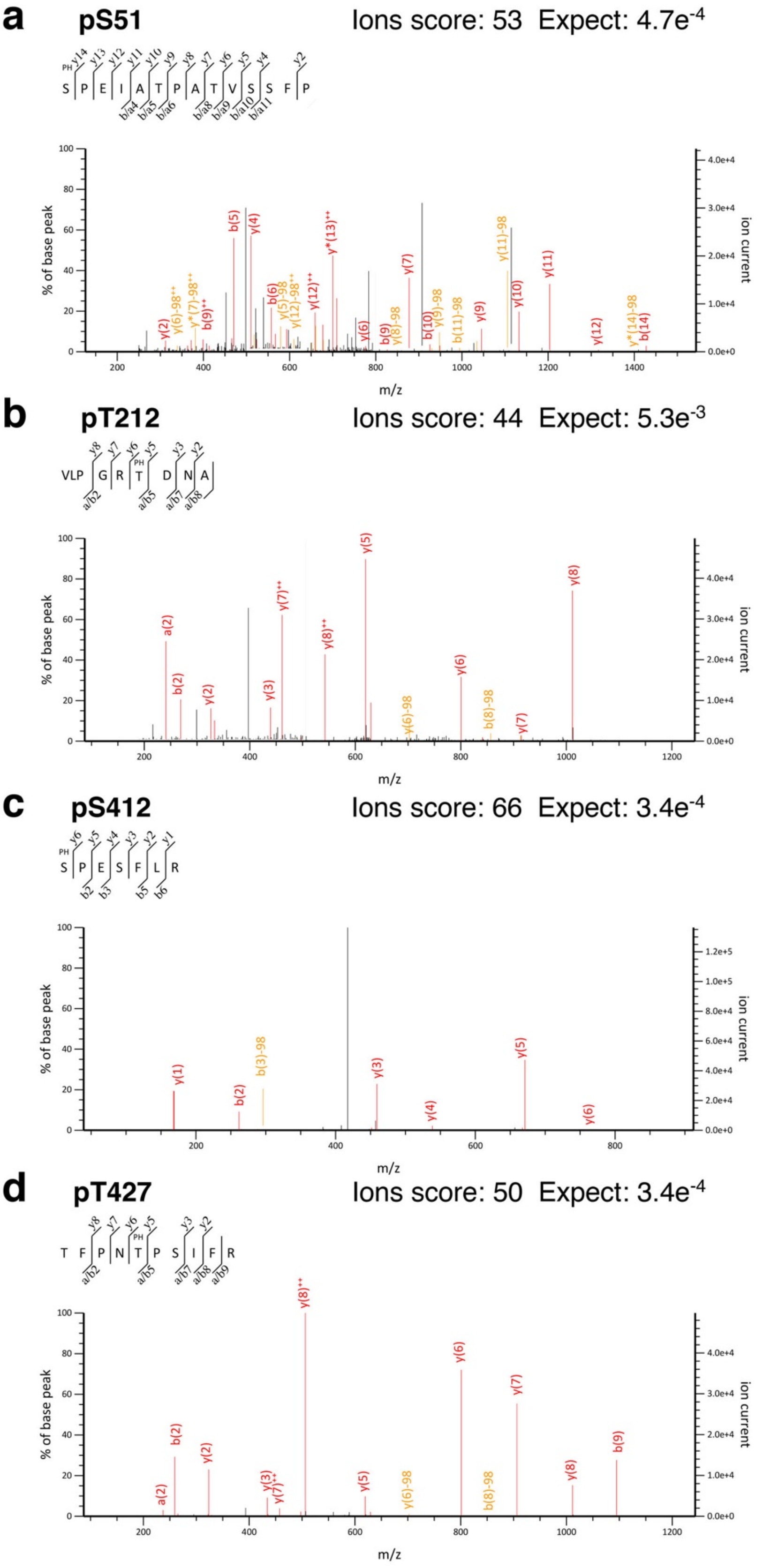


*Continued on next page*

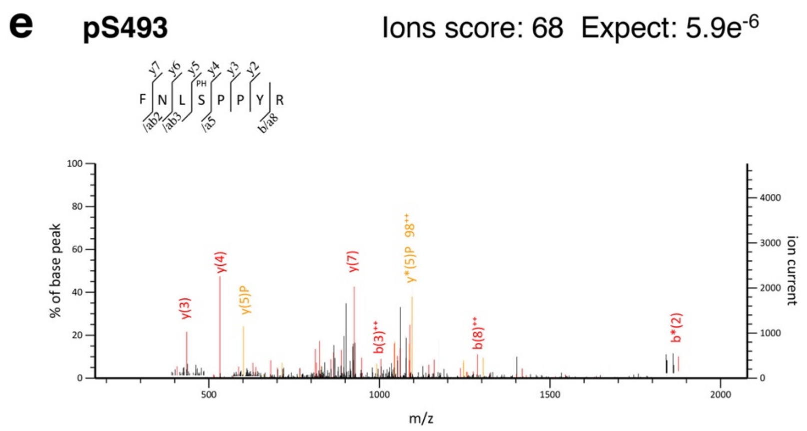


**Supplementary Figure 14.** Identification of phosphorylation sites of MYB3R5 by mass spectrometry. GST-MYB3R5 phosphorylated by CDKA;1-CYCD3;1 complexes was separated by SDS-PAGE and in-gel-digested with trypsin, followed by LC-MS/MS analysis. MS/MS spectra representing phosphorylation at Ser51 (**a**) Thr212 (**b**), Ser412 (**c**), Thr427 (**d**) and Ser493 (**e**) are shown. Ions score indicates values calculated using Mascot software. Peptides with an ions score ≥ 30 and an E-value < 0.05 are shown.

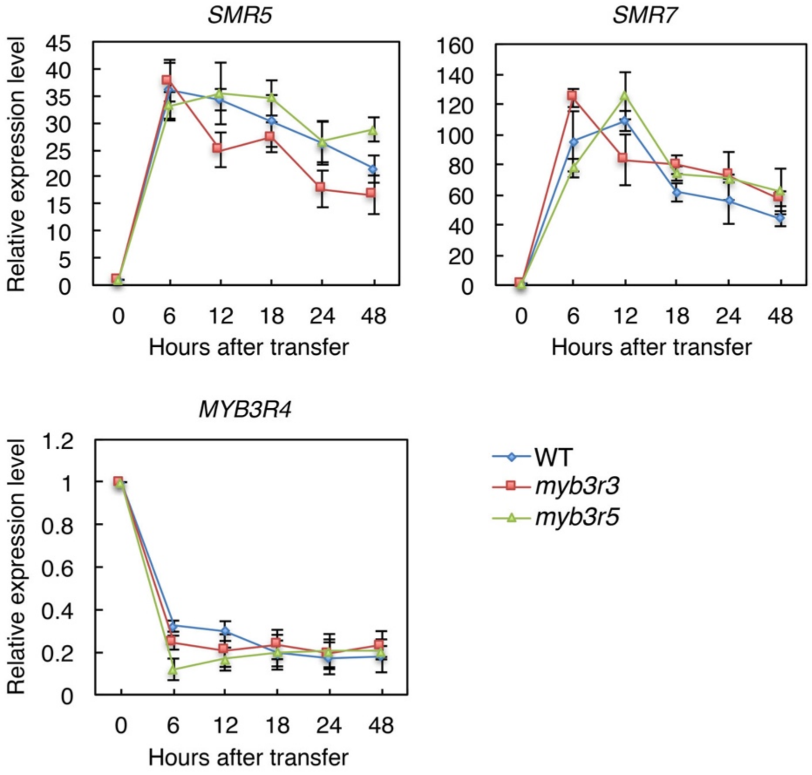


**Supplementary Figure 15.** Expression of *SMR5*, *SMR7* and *MYB3R4* in *myb3r3* and *myb3r5* mutants. Six-day-old seedlings of WT, *myb3r3* and *myb3r5* were transferred to medium containing 2 μM zeocin, and grown for the indicated times. Total RNA was isolated from the region 1 cm from the root tip and subjected to quantitative RT-PCR analysis. The expression levels were normalized to that of *ACTIN2*, and are indicated as relative values, with that for the time of transfer to zeocin-containing medium set to 1. Data are presented as mean ± SD of three biological replicates.

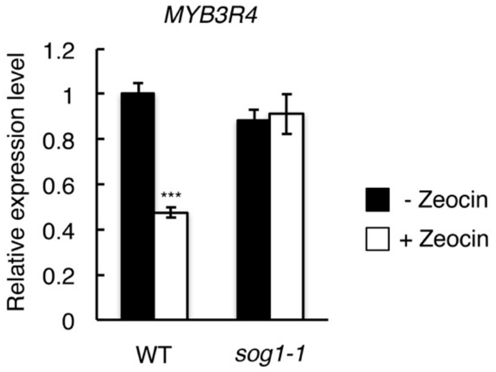


**Supplementary Figure 16.** Expression of *MYB3R4* in the *sog1-1* mutant.

Five-day-old seedlings of WT and *sog1-1* were transferred to medium with or without 6 µM zeocin, and grown for 24 h. Total RNA was isolated from roots and subjected to quantitative RT-PCR analysis. The expression levels were normalized to that of *ACTIN2*, and are indicated as relative values, with that for WT grown in the absence of zeocin set to 1. Data are presented as mean ± SD of three biological replicates. Significant differences from WT grown in the absence of zeocin were determined by Student's *t*-test: ***, $P < 0.001$.

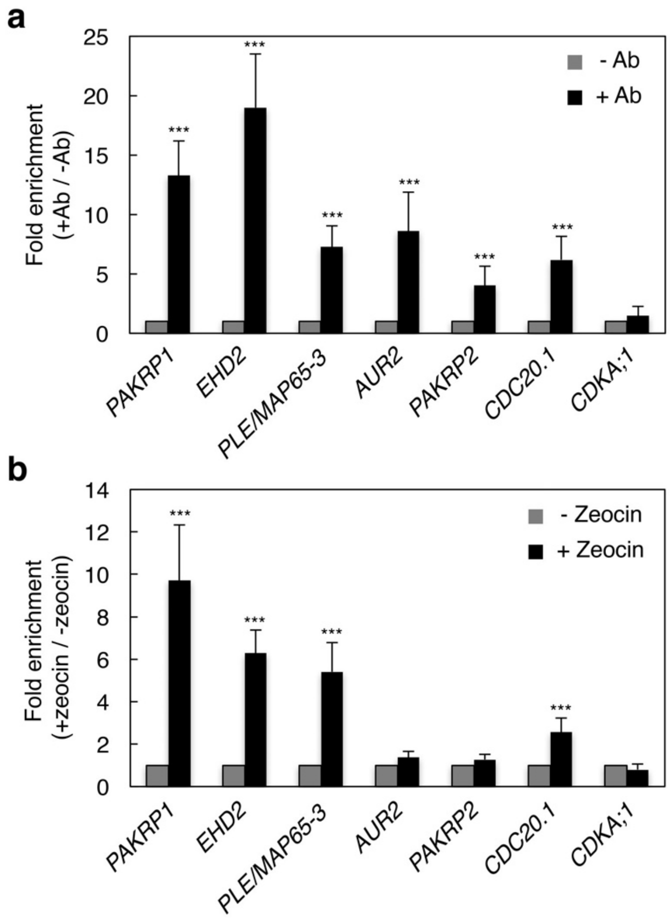


**Supplementary Figure 17.** DNA damage promotes the binding of MYB3R3 to *PAKRP1*, *EHD2* and *PLE/MAP65-3* promoters.

(**a**) ChIP-PCR analysis of G2/M-specific genes. Chromatin bound to MYB3R3 was collected by immunoprecipitation with (+Ab) or without (-Ab) anti-GFP antibodies using roots of 10-day-old *myb3r3* plants carrying *ProMYB3R3::MYB3R3-GFP*. Fold enrichment of each promoter region was determined by normalizing the recovery rate against that of samples immunoprecipitated without the antibody. *CDKA;1* was used as a control that is expressed throughout the cell cycle. Data are presented as mean ± SD of three biological replicates. Significant differences from the control immunoprecipitated without the antibody were determined by Student's *t*-test: ***, $P < 0.001$. (**b**) ChIP-PCR analysis using zeocin-treated roots. Ten-day-old *myb3r3* seedlings carrying *ProMYB3R3::MYB3R3-GFP* were treated with or without 2 μM zeocin for 24 h, and used for ChIP assay. Fold enrichment of each promoter region was determined by normalizing the recovery rate against that of samples without zeocin treatment. Data are presented as mean ± SD of three biological replicates. Significant differences from the control without zeocin treatment were determined by Student's *t*-test: ***, $P < 0.001$.

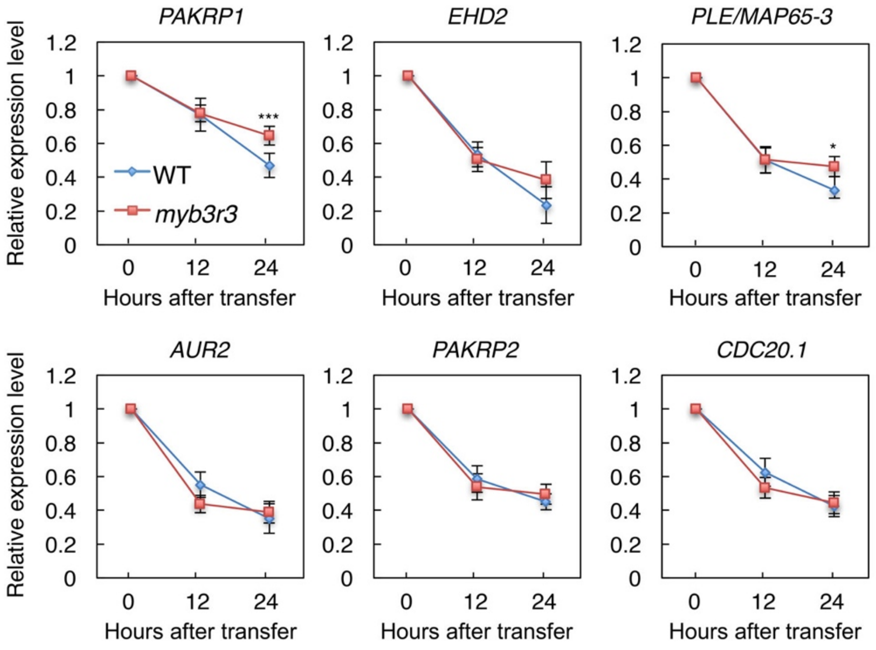


**Supplementary Figure 18.** Quantitative RT-PCR analysis of G2/M-specific genes in *myb3r3*.
Six-day-old seedlings of WT and *myb3r3* were transferred to medium containing 2 µM zeocin, and grown for 12 or 24 h. Total RNA was isolated from a 1-cm region at the root tip. The expression levels were normalized to that of *ACTIN2*, and are indicated as relative values, with that for the time of transfer to zeocin-containing medium set to 1. Data are presented as mean ± SD of three biological replicates. Significant differences from WT were determined by Student's *t*-test: *, $P < 0.05$; ***, $P < 0.001$.

Figure 2b

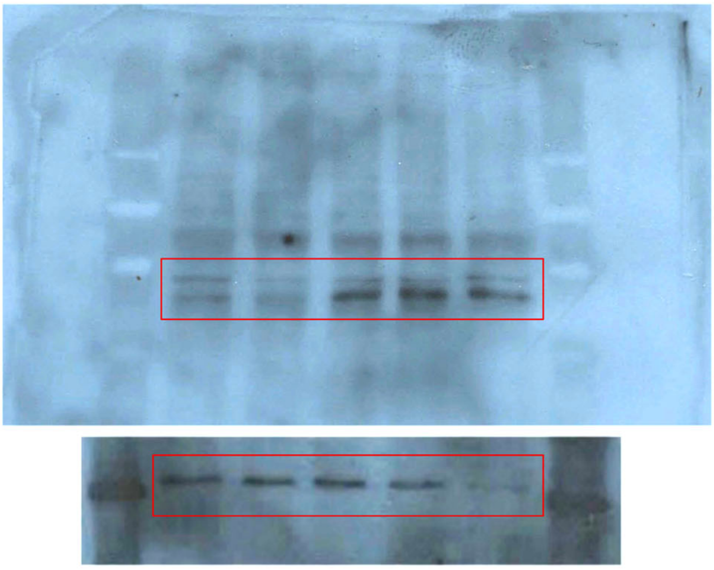

Figure 2d

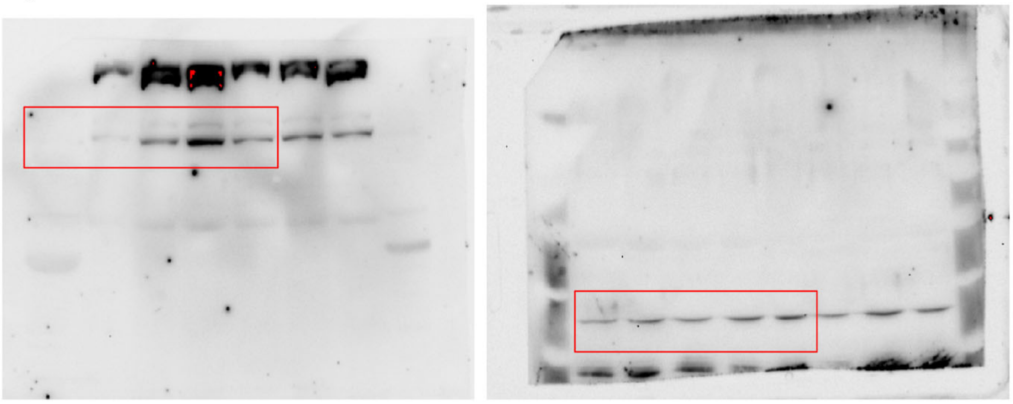

**Supplementary Figure 19.** Uncut blots.

The red sections mark blot results shown in the indicated figures.

**Figure 4a**

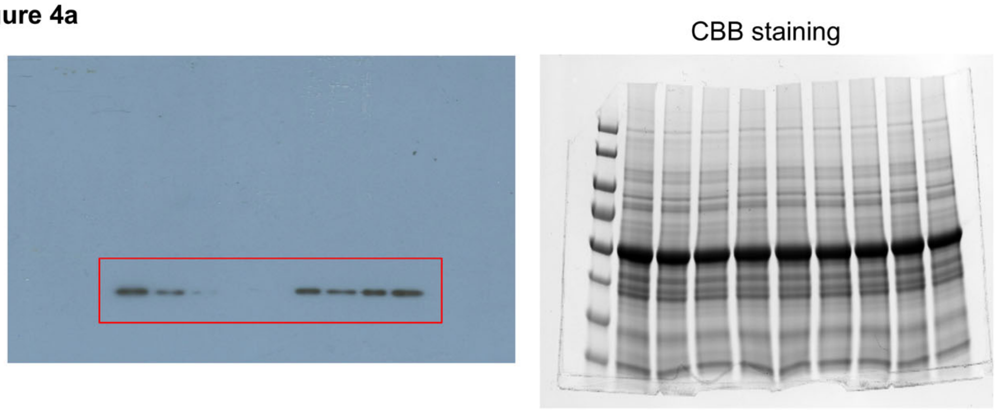


**Figure 4b**

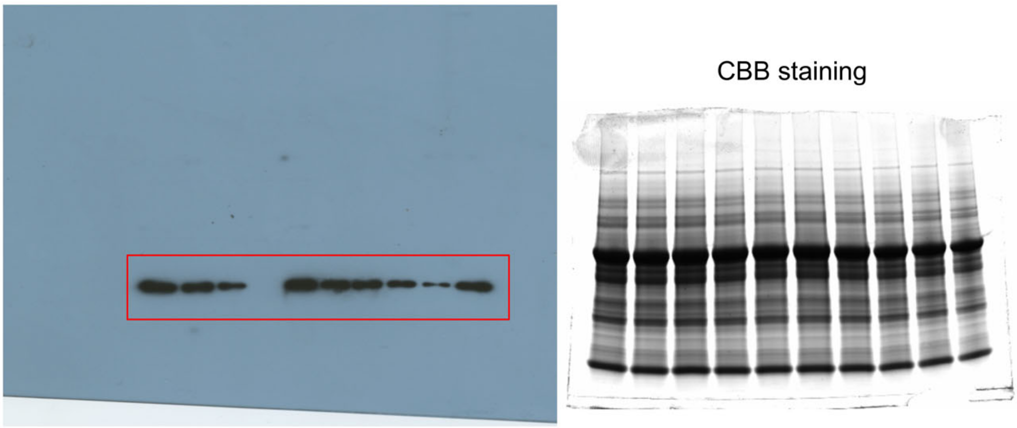


**Figure 4c**

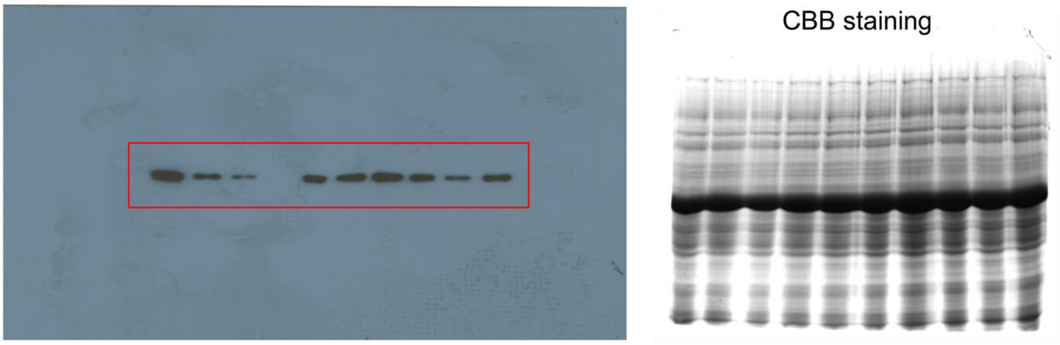


**Supplementary Figure 20.** Uncut blots and gels.

The red sections mark blot results shown in the indicated figures. The results of CBB staining are also shown.

**Figure 5a**

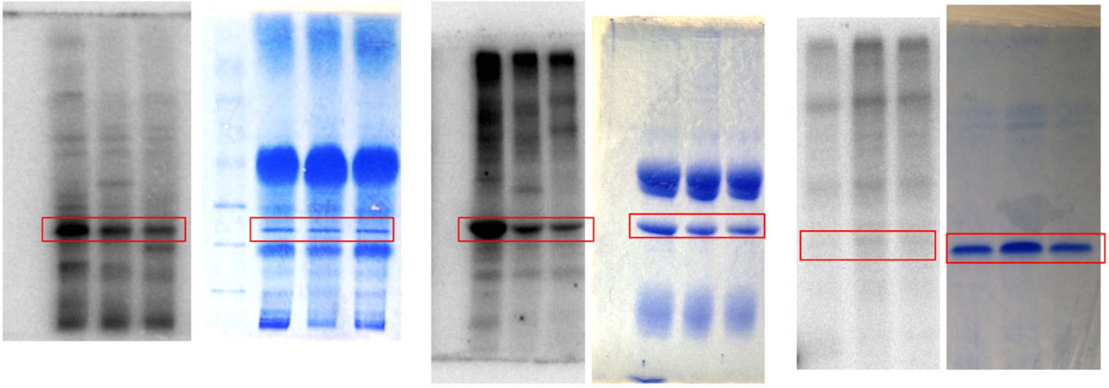

**Figure 5c**

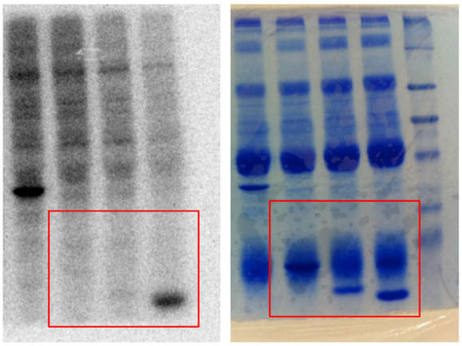

**Figure 5d**

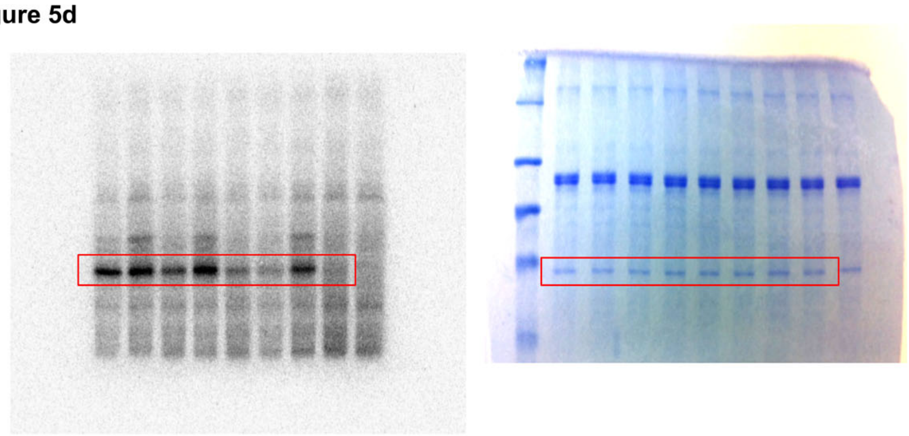

**Supplementary Figure 21.** Uncut blots and gels.

The red sections mark blot and gel results shown in the indicated figures.

**Figure 6b**

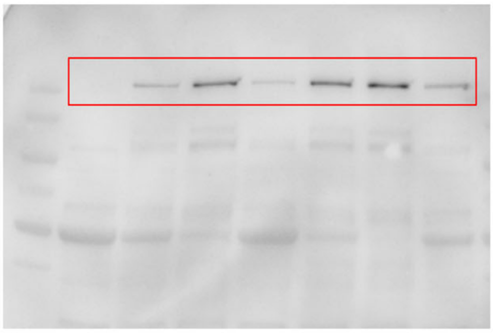

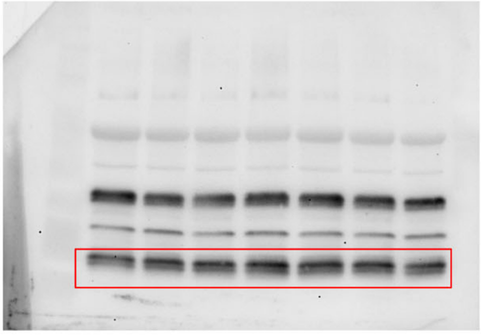

**Supplementary Figure 9b**

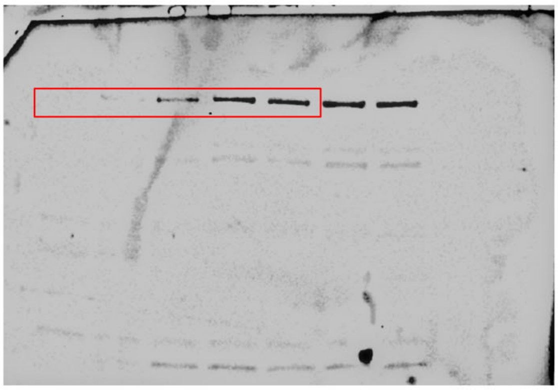

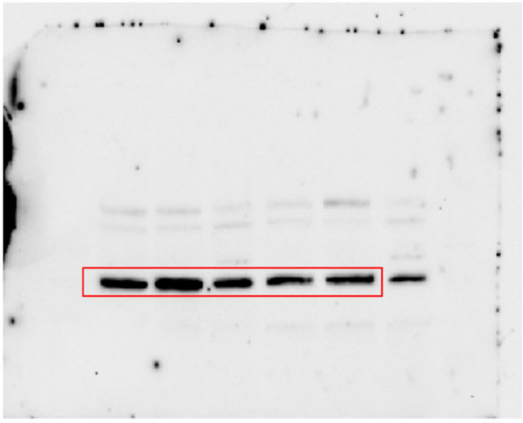

**Supplementary Figure 22.** Uncut blots.
The red sections mark blot results shown in the indicated figures.

## Supplementary Table 1. Primers used for cloning, RT-PCR and ChIP assay

### Cloning

| | | |
|---|---|---|
| *MYB3R3* | 5'-AAAAAGCAGGCTCCATGAGCTCCACTTTTAATCCAGCCG-3'<br>5'-TGTACAAAGTGGCCTTAGCCTAGGAGTTGAGAATCTCCA-3' | Construction of *His-MYB3R3* and *GST-MYB3R3* |
| *MYB3R5* | 5'-AAAAAGCAGGCTCCATGAGCTCAAGTTCGAATCCACCG-3'<br>5'-TGAACAAAGTGGTTCTACACAGGCTTGTCTTCCTCAGAC-3' | Construction of *His-MYB3R5* and *GST-MYB3R5* |
| *R3F1* | 5'-AAAAAGCAGGCTCCATGAGCTCCACTTTTAATCCAGCCG-3'<br>5'-TGTACAAAGTGGCACTATGGTGGTGGTAACCTACCAGTC-3' | Construction of *His-MYB3R3F1* |
| *R3F2* | 5'-AAAAAGCAGGCTCCACAACAACAAGGAATGGTGTTCCT-3'<br>5'-TGTACAAAGTGGTTCTATGGAGTCGAACCGTTACTGTGC-3' | Construction of *His-MYB3R3F2* |
| *R3F3* | 5'-AAAAAGCAGGCTCCAGAAGTCCTGAATCTTACCTGAGA-3'<br>5'-TGTACAAAGTGGCCTTAGCCTAGGAGTTGAGAATCTCCA-3' | Construction of *His-MYB3R3F3* |
| *MYB3R5-genomic* | 5'-AAAAAGCAGGCTCCATGAGCTCCTTCATAAAGAAGATC-3'<br>5'-TGTACAAAGTGGCACAGGCTTGTCTTCCTCAGACGTTTT-3' | Construction of *ProMYB3R5::MYB3R5-GFP* |

### Site-directed mutagenesis

| | | |
|---|---|---|
| *R3 S394A* | 5'-GGTTCGACTCCAAGAGCTCCTGAATCTTACTG-3'<br>5'-CAGGTAAGATTCAGGAGCTCTTGGAGTCGAACC-3' | Alanine substitution at S394 of *MYB3R3* |
| *R3 S394D* | 5'-GGTTCGACTCCAAGAGATCCTGAATCTTACCTG-3'<br>5'-CAGGTAAGATTCAGGATCTCTTGGAGTCGAACC-3' | Aspartic acid substitution at S394 of *MYB3R3* |
| *R3 S461A* | 5'-AATGCTTACAATCTAGCTCCTCCATATCGGATA-3'<br>5'-TATCCGATATGGAGGAGCTAGATTGTAAGCATT-3' | Alanine substitution at S461 of *MYB3R3* |
| *R3 S461D* | 5'-AATGCTTACAATCTAGATCCTCCATATCGGATA-3'<br>5'-TATCCGATATGGAGGATCTAGATTGTAAGCATT-3' | Aspartic acid substitution at S461 of *MYB3R3* |
| *R3 T407A* | 5'-AGAACTTACCCAAACGCACCATCCATATTCAGG-3'<br>5'-CCTGAATATGGATGGTGCGTTTGGGTAAGTTCT-3' | Alanine substitution at T407 of *MYB3R3* |
| *R3 T407D* | 5'-AGAACTTACCCAAACGATCCATCCATATTCAGG-3'<br>5'-CCTGAATATGGATGGATCGTTTGGGTAAGTTCT-3' | Aspartic acid substitution at T407 of *MYB3R3* |

### RT-PCR

| | | |
|---|---|---|
| *ACTIN2* | 5'-GGCTCCTCTTAACCCAAAGGC-3'<br>5'-CACACCATCACCAGAATCCAGC-3' | Real-time PCR of *ACTIN2* |
| *MYB3R3* | 5'-TAAGGTCTTACCCGGCAGGACTGAT-3'<br>5'-CTGA TGAGGTTTGAGCAACTGAACC-3' | Real-time PCR of *MYB3R3* |
| *MYB3R4* | 5'-AATCGCTTGAGAAAGTAGACC-3'<br>5'-AGTAGACAGGACTGGCTTACCG-3'<br>5'-GAGGACTAGTGGCCCTGCAAGACG-3'<br>5'-CACCTGTGGAGGCACTGGACATCAG-3' | Real-time PCR of *MYB3R4*<br><br>Real-time PCR of *myb3r4-3* |
| *MYB3R5* | 5'-GCTAAGGTTTTACCAGGAAGGACTG-3'<br>5'-TTTCCTGAAGAGGTTTGGGTAAGCG-3' | Real-time PCR of *MYB3R5* |
| *KNOLLE* | 5'-CTAATCAGAAGAGTGAAAAAGATG-3'<br>5'-TTCGTAGAAGCCATCTCAAGATC-3' | Real-time PCR of *KNOLLE* |
| *CYCB1;2* | 5'-ATTACGACACCTTGACGTTCTGTC-3'<br>5'-TTTGAGCAGTCCATAATCTCAGAC-3' | Real-time PCR of *CYCB1;2* |
| *EDE1* | 5'-AGAAAGCACAAGCTGAGAGAATG-3'<br>5'-TCCAAAGAAGGAGTTTGTGACTC-3' | Real-time PCR of *EDE1* |
| *IMK2* | 5'-AATCGCTTGAGAAAGTAGACC-3'<br>5'-TTTCTGGTCCTCGAGCGTGAAG-3' | Real-time PCR of *IMK2* |
| *PAKRP1* | 5'-GGTTTGTGCAGTTTCTCCTT-3'<br>5'-GCCTTTGCACGCTGAGCAAA-3' | Real-time PCR of *PAKRP1* |
| *EHD2* | 5'-CCTGAACACACCAGAGGTCG-3'<br>5'-CGTTTATGGGTTTGTCATTG-3' | Real-time PCR of *ATEHD2* |
| *PLE/MAP65-3* | 5'-CCAAGCTCCCAGGTATGGTTGAAG-3'<br>5'-CTGAGTATGTTATACTCCTCAAGC-3' | Real-time PCR of *ATPLE1* |
| *AUR2* | 5'-GGCTCTAGGGTTACTTCTAT-3'<br>5'-CCCTTACAGCATACTCCAAA-3' | Real-time PCR of *ATAUR2* |
| *PAKRP2* | 5'-GCGGGAAGTGATACGCCAGC-3'<br>5'-GGGTTGGTTGGGTTGTTTCC-3' | Real-time PCR of *PAKRP2* |
| *CDC20.1* | 5'-ATATATGGCCCAGAGTCCAGA-3'<br>5'-TCTGTATCATGGGTTTCCTTGTC-3' | Real-time PCR of *CDC20.1* |
| *SMR5* | 5'-TTGCCGGATACCAGCATAC-3'<br>5'-GCGGCTGAAAATATCCCTTC-3' | Real-time PCR of *SMR5* |
| *SMR7* | 5'-CCCACCGGTGTTGAAATG-3'<br>5'-CGTTGTATAAACACCAACTCGAA-3' | Real-time PCR of *SMR7* |

**Supplementary Table 1** (continued)

**ChIP assay**

| | | |
|---|---|---|
| *KNOLLE* | 5'-ATGGACGACAACGGATACATC-3'<br>5'-AGAATTTGATTAGCGATTTGTGA-3' | Detection of the promoter region of *KNOLLE* |
| *CYCB1;2* | 5'-AGCGCATTCCAACTTCGAAGAC-3'<br>5'-AGCGAGCGCTGCTTATGAAGA-3' | Detection of the promoter region of *CYCB1;2* |
| *EDE1* | 5'-AATTTAATCGAGAAAGCAAGAC-3'<br>5'-AAGGAAACTAGCCGTTGAGT-3' | Detection of the promoter region of *EDE1* |
| *IMK2* | 5'-ATTTTGATGCCCCAACGAGTC-3'<br>5'-GGTAAGCGAGAGAAACATGAC-3' | Detection of the promoter region of *IMK2* |
| *CDKA;1* | 5'-ACCATTTTATAAGATAGCAAACCA-3'<br>5'-AATTGAATTTACCTAACCGCA-3' | Detection of the promoter region of *CDKA;1* |
| *PAKRP1* | 5'-GGTTTGTGCAGTTTTCTCCTT-3'<br>5'-GCCTTTGCACGCTGAGCAAA-3' | Detection of the promoter region of *PAKRP1* |
| *ATEHD2* | 5'-CGATGTCGTTTTGCTAACCC-3'<br>5'-GGAAAAAATTTCCAACTCCGG-3' | Detection of the promoter region of *ATEHD2* |
| *ATPLE1* | 5'-AGACCAAAGATCGGCTTAACAC-3'<br>5'-AGGCTTTAGAATCCAGTGCATC-3' | Detection of the promoter region of *ATPLE1* |
| *ATAUR2* | 5'-CGAAATTTATTTAGCGAGTA-3'<br>5'-GGCTTCTAGAATTATATGGT-3' | Detection of the promoter region of *ATAUR2* |
| *PAKRP2* | 5'-CCTCACGTGCCACGTGGGAT-3'<br>5'-CAGAACGCATTCACATGTTTT-3' | Detection of the promoter region of *PAKRP2* |
| *CDC20.1* | 5'-CCTCTTTTTTTTGGGTGCAT-3'<br>5'-GCGAAAAAACACTTGGTATT-3' | Detection of the promoter region of *CDC20.1* |
